# Protective Antiviral Immunity Conferred by a Nonintegrative Lentiviral Vector-Based Vaccine

**DOI:** 10.1371/journal.pone.0003973

**Published:** 2008-12-19

**Authors:** Frédéric Coutant, Marie-Pascale Frenkiel, Philippe Despres, Pierre Charneau

**Affiliations:** 1 Laboratoire de Virologie Moléculaire et Vectorologie, Institut Pasteur, Paris, France; 2 Unité des Interactions Moléculaires Flavivirus-Hôtes, Institut Pasteur, Paris, France; Karolinska Institutet, Sweden

## Abstract

Lentiviral vectors are under intense scrutiny as unique candidate viral vector vaccines against tumor and aggressive pathogens because of their ability to initiate potent and durable specific immune responses. Strategies that alleviate safety concerns will facilitate the clinical developments involving lentiviral vectors. In this respect, the development of integration deficient lentiviral vectors circumvents the safety concerns relative to insertional mutagenesis and might pave the way for clinical applications in which gene transfer is targeted to non-dividing cells. We thus evaluated the potential use of nonintegrative lentiviral vectors as vaccination tools since the main targeted cell in vaccination procedures is the non-dividing dendritic cell (DC). In this study, we demonstrated that a single administration of nonintegrative vectors encoding a secreted form of the envelope of a virulent strain of West Nile Virus (WNV) induces a robust B cell response. Remarkably, nonintegrative lentiviral vectors fully protected mice from a challenge with a lethal dose of WNV and a single immunization was sufficient to induce early and long-lasting protective immunity. Thus, nonintegrative lentiviral vectors might represent a safe and efficacious vaccination platform for the development of prophylactic vaccines against infectious agents.

## Introduction

Prevention of infectious diseases through vaccine development is one of the greatest achievements of modern medicine. Nonetheless, considerable challenges remain for the development of new vaccines combining efficiency with enhanced safety profiles. Recently, human immunodeficiency virus (HIV)-1–derived lentiviral vectors have emerged as very promising vaccination tools. These vectors elicit both specific cytotoxic and strong humoral immune responses in several animal models [1], [2], [3], [4], [5]. Immune responses elicited by lentiviral vectors are more efficient than those induced by conventional viral vector vaccines [6], [7], [8]. These properties rely in part on the ability of these vectors to mediate more efficient gene transfer into dendritic cells (DC) than other vaccinal vectors such as for instance the widely used adenoviral vectors [9]. This major advantage allows a sustained expression and endogenous presentation of tumoral or viral Ags by transduced DC and subsequent activation of more potent and persistent specific immune responses [3], [10], [11], [12], [13]. Moreover, problems of vector-specific immunity are largely reduced with the use of LV because of the absence of pre-existing immunity in humans.

Although lentiviral vector-based vaccines have been shown to elicit unparallel specific and protective immunity against tumor and infectious agents, their transition as vaccinal vectors from preclinical evaluation to clinical development is hindered by their status of integration-competent viral vectors and potential problems linked to the non-specific integration of the transgene in the genome of the transduced cells. However, several arguments point out that lentiviral vectors possess a much safer profile than other retroviral vectors, especially when they are applied to the field of vaccination. Firstly, in a vaccination scenario based on direct injection of Ag-encoding lentiviral vectors, transduced cells that express the relevant Ag become targets of the elicited immune response and are eliminated within a few weeks from the vaccinated organism [14]. Secondly, the deletion in the 3′ LTR of the viral promoter and enhancer sequences in self-inactivating lentiviral vectors limits the likelihood of endogenous promoter activation [15]. Thirdly, although many lymphocytes of HIV-infected patients carry a large number of transcriptionally active HIV integrated proviruses, there is no evidence to suggest that integration of lentiviruses would cause oncogenesis. Consistently, a recent study has provided evidences that lentiviral vector transduction, even at high integration loads do not accelerate tumorigenesis in a tumor-prone murine model [16]. Nevertheless, investigations to confirm the safety of lentiviral vectors or elaboration of novel strategies bypassing these safety considerations need to be actively pursued. In this context, the development of lentiviral vectors that allows a targeted integration or does not integrate at all would provide an important step toward the development of fully safe vector-based vaccines. The last strategy can be easily achieved with the use of integration-deficient lentiviral vectors carrying a defective integrase mutated in residues critical for the integration of viral DNA into the host genome. One of the most well-studied mutation is the D64V substitution in the catalytic domain of the enzyme, which has been shown to block the DNA cleaving and joining reactions of the integration step [17]. The incorporation of this mutation in pseudotyped HIV-1 particles or in lentiviral vector particles leads to only barely detectable integration events and accumulation of extrachromosomal non-replicating circular genomes in the nucleus of the transduced cells [18]. These circular genomes are diluted through cell division but support transcription by the cellular machinery, allowing stable transgene expression in non-dividing cells. As such, lentiviral vectors defective for integration could supersede their integrating counterparts in clinical purposes that target postmitotic cells. This notion was supported by recent studies demonstrating the efficiency of nonintegrative lentiviral vectors to transduce primary cells such as neuronal cells [19], muscle cells [20] or retinal cells and to rescue representative models of retinal degeneration [21].

In this study, we asked whether integration-deficient lentiviral vectors could initiate a specific and protective immune response. We hypothesized that the induction of Ag specific immune responses in vaccine strategies with lentiviral vectors should not require vector integration, since targeted cells are non-dividing DC. To test this hypothesis, we firstly evaluated the ability of these vectors to transduce *in vitro* different subpopulations of DC. We next investigated whether immunization with nonintegrative lentiviral vector particles was efficient to stimulate a specific and protective humoral immune response against West Nile Virus (WNV), a flavivirus responsible for the largest recognized epidemic of neuroinvasive human disease in North America.

We show that integration-deficient lentiviral particles are not only efficient in transducing DC but also that a single immunization with these vectors coding for the secreted form of the WNV envelope can prime humoral Ag-specific responses and confers complete protection against lethal challenge.

## Results

### Transduction of non-dividing cells with lentiviral vectors deficient for integration results in high transgene expression levels

To test the hypothesis that integration deficient lentiviral vectors could be efficient tools to deliver Ags to non-dividing antigen-presenting cells such as DC, we initially evaluated their transduction efficiency in growth-arrested cells. For this purpose, HeLa cells treated with aphidicolin, a specific inhibitor of cell cycle, were exposed to increasing doses of nonintegrative or integrative lentiviral vectors encoding eGFP and transduction efficiency was determined by flow cytometry. It is important to note that, as previously described [21], the titers of integrative and nonintegrative lentiviral vectors were similar according to p24 content and quantitative PCR titration as described in Material and Methods. A similar expression kinetics experiments in dividing HeLa cells using the integrase defective (D64V) vector, led to progressive loss of GFP transgene expression to undetectable levels, as previously described [20], [21], confirming the non integrated status of the vector genome (data not shown).

**Figure 1:**
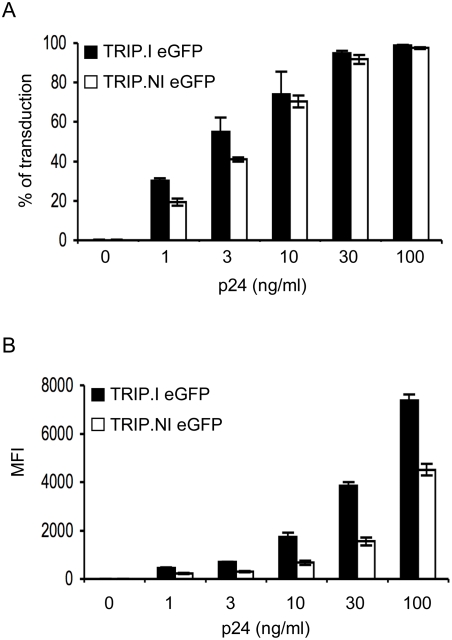
Efficient transduction of non-dividing cells with lentiviral vectors defective for integration. Aphidicolin-treated HeLa cells were transduced with graded doses (1–100 ng of p24 antigen) of eGFP-integrative vectors (TRIP.I eGFP) or eGFP-nonintegrative vectors (TRIP.NI eGFP). FACS analysis of the percentage of GFP positive cells (A) and the MFI (B) at 48 hours post-transduction is shown.

As shown in Figure 1A, integration-deficient lentiviral vectors transduced non-dividing cells with high efficiency and in a dose dependent manner. Moreover, analysis of the percentage of eGFP positive cells revealed marginal differences in the capacities of transduction of integration-deficient vectors compared with integration-proficient vectors. Transduction with nonintegrative vectors yielded also high levels of expression of the transgene (Fig. 1B), although significantly lower by a 2-fold factor compared to integrative lentiviral vectors.

### Nonintegrative lentiviral vector transduction leads to effective antigen expression both in conventional and in plasmacytoid dendritic cells

**Figure 2:**
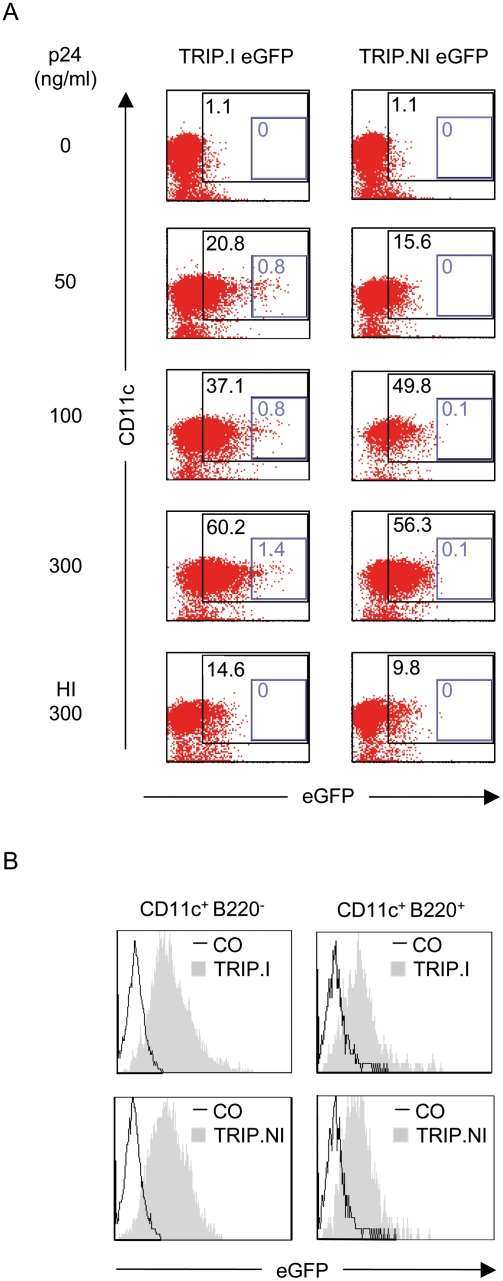
Lentiviral vector transduction leads to effective antigen expression both in conventional dendritic cells (cDC) and in plasmacytoid DC (pDC). (A) Dose-response transduction experiments with eGFP-integrative vectors (TRIP.I eGFP) or eGFP-non integrative vectors (TRIP.NI eGFP), heat-inactivated (HI) or not. On day 6 of differentiation, FL-DC were exposed to vector particles for 48 hours and transduction of CD11c positive cells was assessed by measuring eGFP expression by flow cytometry. Numbers indicate the percentage of CD11c^+^ cells expressing eGFP. Blue gates delineate a DC population expressing high levels of eGFP. (B) Transduction of pDC and cDC by lentiviral vectors. Expression of eGFP by cDC (CD11c^+^ B220^−^) and pDC (CD11c^+^ B220^+^) is shown. Thin lines, control cells; filled profiles, FL-DC transduced with 300 ng/ml of vector particles.

We next studied the ability of integration-deficient lentiviral vectors to transduce DC. Murine DC are categorized into conventional (cDC) (CD11c^+^ B220^−^) and plasmacytoid (pDC) (CD11c^+^ B220^+^) and both these DC subtypes are able to stimulate Ag-specific immune responses [22]. We then investigated the transduction of bone marrow-derived DC differentiated in presence of Flt3L (FL-DC), which allows the generation of large numbers of pDC and cDC. FL-DC were exposed to increasing doses of integration-deficient or –proficient eGFP-encoding vectors. As shown in Figure 2A, both integrative and nonintegrative lentiviral vectors were capable of transducing FL-DC with maximal transduction of efficiency of 60% and 56% respectively. Interestingly, we observed that transduction with integrative vectors led to a small proportion of DC expressing high levels of eGFP, whereas transduction experiments with nonintegrative vectors did not (Blue gates, 1.4% with TRIP.I eGFP and 0.1% with TRIP.NI eGFP). To rule out the possibility of pseudo-transduction conferred by residual eGFP proteins included in the particles, we also evaluated the percentage of transduced DC after exposure to particles submitted prior to a heat-treatment, which has been shown to abrogate the transduction capabilities of lentiviral vectors on different cell types. As expected, the heat-treatment decreased drastically the percentage of eGFP positive cells. The same level of reduction was also observed in growth-arrested cells transduced with nonintegrative lentiviral vectors in the presence of Nevirapin, a specific inhibitor of the reverse transcriptase (data not shown). Thus, the GFP fluorescence resulted from *de novo* transcription of the transgene rather than from pseudotransduction ofencapsidated GFP proteins.

We next gated on CD11c^+^ B220^+^ and CD11c^+^ B220^−^ to evaluate the capacity of lentiviral vectors to transduce each DC subset. As shown in Fig. 2B, not only FL-derived cDC but also FL-derived pDC could be efficiently transduced with lentiviral vectors, regardless of their integration proficiencies. Thus, efficient transduction can be achieved in different subpopulations of DC with integration-deficient lentiviral vectors, although levels of expression of the transgene are lower compared to integrative vectors-transduced cells.

### Nonintegrative lentiviral vectors induce the production of Ag-specific antibodies

**Figure 3:**
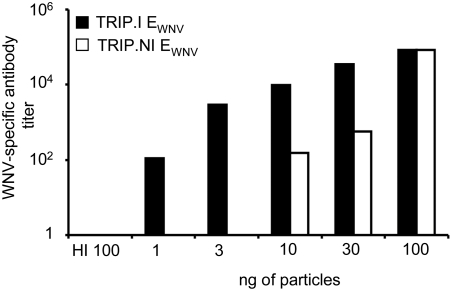
A single dose of nonintegrative lentiviral vector coding for E_WNV_ elicits a strong and specific antibody response. Groups of adult mice (n = 6) were immunized intraperitoneally with graded doses (1–100 ng of p24 antigen) of TRIP.NI E_WNV_ or TRIP.I E_WNV_ particles. Control mice were inoculated with 100 ng of heat-inactivated (HI) particles. After 21days, pooled sera (6 mice per group) were assessed for the presence of WNV-specific antibodies. Data are representative of three independent experiments.

Taking into account that integration-deficient lentiviral vectors could allow efficient expression of a foreign gene in professional antigen-presenting cells, we next explored their abilities to mount a specific immune response. In a recent study, we have designed integrative lentiviral vectors coding for a secreted form of WNV envelope (E_WNV_) which possesses neutralizing epitopes and we have demonstrated that these vectors could stimulate a massive production of anti-WNV antibodies [4]. To investigate the ability of nonintegrative lentiviral vectors to initiate a B cell response and to compare the strength of the immune response elicited by lentiviral vectors defective or not for integration, groups of C57/Bl6 mice (n = 6) were immunized with various doses of integrative or nonintegrative vectors coding for E_WNV_ ranging from 1 to 100 ng of p24 antigen per mouse (corresponding to 1×10^5^ to 1×10^7^ transduction units). As a control, mice were inoculated with 100 ng of nonintegrative E_WNV_ vector particles inactivated by heating to abrogate their transduction capacities. Three weeks after immunization, pooled sera collected from each group of immunized mice were tested for the presence of total anti-WNV antibodies. As expected, immunizations with 100 ng of heat-inactivated lentiviral vectors (HI 100) were not followed by the production of antibodies (Fig. 3). By contrast, mice immunized with a dose as low as 10 ng of nonintegrative E_WNV_ vectors displayed detectable levels of anti-WNV antibodies and immunizations with 100 ng of this vector induced a massive secretion of anti-WNV Ig with a mean titer reaching 8×10^4^. As shown in Fig. 3, integrative lentiviral vectors elicited a higher immune response than nonintegrative vectors at doses below 30 ng, but vaccinations with 100 ng of either vector led to an equivalent intense humoral immune response.

Taken together, these results demonstrated that a single immunization with nonintegrative lentiviral vectors was sufficient to elicit a humoral specific immune response with a strength comparable to that obtained with integrative vectors, above a threshold dose of particles.

### Immunization with nonintegrative lentiviral vectors confers early protection against WNV challenge

**Figure 4:**
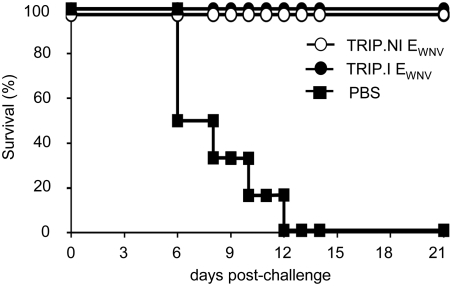
Rapid protection against WNV infection conferred by nonintegrative lentiviral vector immunization. Six mice/group were vaccinated with 100 ng of TRIP.NI E_WNV_ or 100 ng of TRIP.I E_WNV_. A control group of mice inoculated with phosphate-buffered saline (PBS) was included. One week after the vaccination, mice were challenged with 1,000 i.p. LD_50_s of WNV strain IS-98-ST1. Survival was recorded for 21 days. Data are representative of three independent experiments.

We have previously shown that integrative lentiviral vectors conferred an early protective immunity against a WNV challenge, a critical feature in the context of a WNV outbreak. To determine if the immune response elicited by integration-deficient lentiviral vectors could also lead to a rapid protection, mice were immunized with 100 ng of nonintegrative E_WNV_ vector particles and challenged 7 days later with 10,000 FFU of the highly virulent WNV strain IS-98-ST1 (one thousand times the dose required to kill 50% of infected animals). We also included in this challenge experiment a group of mice immunized with 100 ng of integrative E_WNV_ lentiviral vectors as a positive control of protection and another group of mice inoculated with D-PBS as a negative control. As expected, all mice that received D-PBS died within 12 days post-challenge (Fig. 4). In contrast, all mice immunized with a single dose of nonintegrative E_WNV_ lentiviral vectors were protected from the challenge, as were mice immunized with integrative vectors. Moreover, mice protected from WNV challenge did not develop any clinical signs of illness during the 3-weeks post-challenge observation period. These results demonstrated that an early protective immunity against WNV was achieved with a single administration of lentiviral vector defective for integration.

### Nonintegrative lentiviral vector immunization induces long-lasting protection

**Figure 5:**
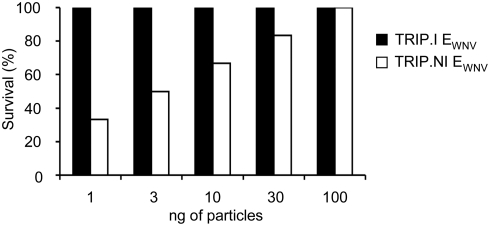
Efficient long-term protection against WNV infection by nonintegrative lentiviral vector-based vaccine. Two months post-immunization with graded doses (1–100 ng of p24 antigen) of TRIP.NI E_WNV_ or TRIP.I E_WNV_ particles, groups of mice (6 mice per group) were inoculated with 1,000 i.p. LD_50_s of WNV strain IS-98-ST1. Survival was recorded for 21 days. Data are representative of three independent experiments.

As demonstrated earlier, immunization of mice with integrative lentiviral vectors coding for E_WNV_ resulted in the establishment of long-term protective immunity against WNV challenge [4]. To evaluate the duration of the protective immunity elicited by nonintegrative E_WNV_ lentiviral vectors and the minimum dose of particles required to induce long-term protection, mice were immunized with different amounts of particles (1, 3, 10, 30 and 100 ng of p24 antigen) and were challenged 2-months post-immunization. As shown in Figure 5, there was a dose-dependent relationship between the quantity of nonintegrative E_WNV_ vector particles administrated and the degree of protection, with a full protection achieved at a dose of 100 ng of vaccine particles injected. In contrast, a 100% protection was achieved after a single injection of integrative E_WNV_ vector particles regardless the dose. Thus, long-lasting immunity against WNV infection can be achieved with a nonintegrative lentiviral vector-based vaccine.

## Discussion

We have demonstrated for the first time that safe nonintegrative lentiviral vectors are not only effective at transducing different subpopulations of DC but also that a single immunization with these vectors was sufficient to afford complete protective immunity against a lethal viral challenge in mice. Transduction efficiency of both plasmacytoid and conventional DC with nonintegrative viral particles was dose dependent and led to the same levels of transduction than those obtained with integrative particles. Although transduction of both growth-arrested cells and DC with nonintegrative lentiviral vectors led to high levels of expression of the transgene, the levels were approximatively 2 fold lower than that achieved with integrative lentiviral vectors.

Immunizations of mice with a single dose of 100 ng of nonintegrative lentiviral vectors coding for a secreted form of the envelope of WNV induced an equivalent level of specific WNV antibodies to that obtained with the same dose of integrative vectors with a mean titer reaching 8×10^4^. However, dose-response experiments revealed that the minimal dose required for the induction of a B cell response was lower with TRIP.I particles compared to the TRIP.NI particles. One possible explanation for this result could be related to the ability of integrative lentiviral vectors to generate a more sustained production of Ag in transduced DC since, theoretically, high expression levels of the Ag in the DC could favor a more sustained presentation of antigenic peptides and may thus explain why low doses of integrative vector particles were sufficient to elicit a specific immune response. An alternative or complementary explanation is that VSV-G pseudotyped particles have a large cellular tropism and thus, may transduce at the site of injection other cell types than DC, including dividing cells. This could result in a more sustained expression of the Ag in vaccination experiments with integrative vector particles. Which cell types are transduced after *in vivo* injections of lentiviral vector particles and to what extend they are involved in the magnitude of the immune response elicited by integrative and nonintegrative lentiviral vectors is the subject of ongoing research.

An important result of our study was the demonstration that vaccination with a single dose of 100 ng of nonintegrative E_WNV_ particles was sufficient to elicit a very early, long-lasting and fully protective immunity against a challenge with a lethal dose of WNV. Kinetic challenge experiments on vaccinated mice revealed that nonintegrative vector-based vaccines conferred a protective immunity as early as one week after a single injection of particles. Although the exact mechanisms involved in this early protection are not fully understood, we have detected specific WNV antibodies one week after immunizations with nonintegrative and integrative vector particles (data not shown). We have previously shown that this early wave of antibodies were exclusively composed of specific IgM, which have been shown to completely protect mice against WNV infection [4], [23]. Intriguingly, low doses (1 and 3 ng of particles) of nonintegrative lentiviral vectors were able to confer a partial long-lasting protective immunity against WNV challenge (Fig. 5), although no WNV-antibodies were detected in the sera of animals 3 weeks after injection (Fig. 3). Even if it is widely accepted that the humoral immune response is an essential component of protective immunity, these unexpected results suggest that cellular immunity could also play a partial role in the establishment of protection against WNV. Consistent with this hypothesis, two recent studies have identified CTL epitopes in the envelope and CTL directed against these epitopes conferred partial protection against WNV challenge. Whether a T cell response could be mounted by nonintegrative lentiviral vectors and could contribute to protection against WNV challenge remains to be determined. However, data obtained by us and others [24] in another model of antigenic vaccination support the concept that nonintegrative lentiviral vectors can also prime CD8+ T cells.

Until now, potential applications of lentiviral vectors in the field of vaccinology were mainly restricted to therapeutic interventions against fatal diseases such as advanced cancers [25]. Lentiviral vectors deficient for integration offer great potential for therapeutic vaccination but the demonstration that they can elicit effective protective immunity opens also for the first time the possibility of using lentiviral vectors in large-scale prophylactic vaccinations. Indeed, this new generation of lentiviral vector-based vaccines alleviates concerns of potential problems associated with non specific integration of a transgene since residual integration events with these lentiviral vectors mutants has been shown to be barely detectable and are due to non-homologous recombination events [18]. These rare integration events may also occur with other transient gene delivery methods, such as adenoviral vectors or nonviral plasmid-based vaccines. Moreover, nonintegrative lentiviral vectors preserve many favourable features of conventional integrative lentiviral vectors, including vector stability, high production yields and infectivity. Finally, a single injection of nonintegrative lentiviral vectors can be sufficient to induce a fully protective immunity against pathogens, as illustrated in our study. This represents a major advantage in terms of cost-effectiveness, allowing mass vaccination even in developing countries.

Even if further works are needed to explore the efficacy of nonintegrative lentiviral vectors especially in the field of specific cellular immune responses, we envision that nonintegrative- based vaccine strategy could represent a very promising platform for the development of vaccines with fewer safety concerns and potent protection immunity.

## Materials and Methods

### Cell culture and virus preparations

Hela cells, Human 293T cells and African green monkey kidney Vero cells were cultured in Dulbecco modified Eagle medium (DMEM) supplemented with 10% (Hela cells, 293T cells) or 5% (Vero cells) heat-inactivated fetal calf serum (FCS), penicillin, streptomycin and Glutamax (GIBCO). WNV strain IS-98-ST1 (GenBank accession number AF 481 864) [26], a closely related variant of NY99 strain [27], [28], [29], was propagated in mosquito *Aedes pseudoscutellaris* AP61 cell monolayers. Purification in sucrose gradients and virus titration on AP61 cells by focus immunodetection assay using anti-WNV hyperimmune mouse ascitic fluid were performed as previously described [30], [26], [31]. Infectivity titers were expressed as focus forming units (FFU).

### Lentiviral vector production

The TRIP E_WNV_ and TRIP GFP vector plasmids were constructed as previously described [4]. Vector particles were produced by transient calcium phosphate co-transfection of 293T cells with the vector plasmid pTRIP/sEwnv, a VSV-G envelope expression plasmid (pHCMV-G) and an encapsidation plasmid (p8.74 or pD64V for the production of integration-proficient or integration-deficient vectors respectively) as previously described [32]. Quantification of the p24 antigen content of concentrated vector particles was performed with a commercial HIV-1 p24 enzyme-linked immunosorbent assay (ELISA) kit (Perkin Elemer Life Sciences). Vector titers of TRIP.I and TRIP.NI particles were determined by transducing HeLa cells treated with aphidicolin and performing a quantitative PCR as previously described [4]. The titers of integrative and nonintegrative lentiviral vectors were similar according to p24 content and quantitative PCR measured in growth-arrested cells.

### Preparation of bone marrow-derived dendritic cells

Bone marrow cells were isolated by flushing femurs and tibiae with RPMI supplemented with 10% FCS. Cells were then passed through a 45-µm cell strainer, centrifuged and resuspended in IO Test 3 lysing solution (Beckman Coulter) and incubated at 4°C for 5 min to lyze red blood cells. The cells were centrifuged and cultured for 8 days at 1×10^6^ cells/ml in culture medium consisting of RPMI with 10% FCS, L-glutamine, penicillin, streptomycin, 1 mM sodium pyruvate, 10 mM HEPES, and 50 µM 2-mercaptoethanol supplemented with 100 ng/ml of recombinant mouse FLT3 ligand (R&D Systems).

### Transduction experiments and flow cytometry analysis

For transduction experiments on non-dividing cells, Hela cells were seeded in 48 wells plates at 40,000 cells/well in the presence of 8 µM of aphidicolin (Sigma). Cells were transduced 24 hours after the aphidicolin block, which was replenished in the medium at the time of transduction. At 2 days post-transduction, cells were harvested and eGFP expression was analyzed by flow cytometry. For DC transduction experiments, FLT3L-generated-bone marrow-derived DC (FL-DC) were transduced at day 6 of the differentiation. At 2 days post-transduction, FL-DC were harvested and resuspended in PBS with 2% FCS and 0.01% sodium azide (staining buffer). Cells were stained with an APC-conjugated anti-CD11c antibody (BD biosciences) and a PerCP-conjugated anti-B220 antibody (BD biosciences), washed twice and analyzed by flow cytometry on a FACSCalibur (BD biosciences, Franklin Lakes, NJ).

### Mice immunization and WNV challenge

All animal experiments were conducted in accordance with the guidelines of the Office Laboratory of Animal Care at the Pasteur Institute. Six-week-old C57/Bl6 mice were intraperitoneally (i.p.) inoculated with varying doses of TRIP.E_WNV_ vector particles in 0.1 ml Dulbecco's phosphate-buffered saline (DPBS; pH 7.5) supplemented with buffered 0.2% bovine serum albumin (DPBS/0.2% BSA, Sigma). WNV challenge was performed by i.p. inoculation of neurovirulent WNV strain IS-98-ST1 (i.p. LD 50 = 10 focus-forming units (FFU)) as previously described [30], [26]. The challenged mice were monitored daily for signs of morbidity or mortality, for up to 21 days.

### Measurement of serum antibody responses

Mice were bled via the periorbital route and serum samples were heat-inactivated 30 min at 56°C. Anti-WNV antibodies were detected by ELISA, by use of microtitration plates coated with sucrose-purified WNV IS-98-ST1. Peroxydase goat anti-mouse immunoglobulin (H+L) (Jackson Immuno Research) was used at a 1:4,000 dilution as secondary antibodies. The endpoint titer was calculated as the reciprocal of the last dilution eliciting twice the optical density of sera from nonimmunized mice.
